# Ultrafast Carrier Relaxation Dynamics in Quantum Confined Non-Isotropic Silicon Nanostructures Synthesized by an Inductively Coupled Plasma Process

**DOI:** 10.3390/ma13194267

**Published:** 2020-09-25

**Authors:** Stefano Ponzoni, Sonia Freddi, Marta Agati, Vincent Le Borgne, Simona Boninelli, Richard Dolbec, My Ali El Khakani, Stefania Pagliara, Paola Castrucci

**Affiliations:** 1 I-Lamp and Dipartimento di Matematica e Fisica, Università Cattolica del Sacro Cuore, via dei Musei 41, 25121 Brescia, Italy; stefano.ponzoni@tu-dortmund.de (S.P.); sonia.freddi@unicatt.it (S.F.); stefania.pagliara@unicatt.it (S.P.); 2 Dipartimento di Fisica, Università degli Studi di Roma Tor Vergata, via della Ricerca Scientifica 1, 00133 Roma, Italy; 3 Dipartimento di Fisica e Astronomia, Università degli Studi di Catania, Via S. Sofia 64, 95123 Catania, Italy; marta.agati@ct.infn.it; 4 Institut National de la Recherche Scientifique, Centre-Énergie, Matériaux et Télécommunications, 1650 Blvd. Lionel-Boulet, Varennes, QC J3X-1S2, Canada; vleborgne@gkmconsultants.com (V.L.B.); elkhakani@emt.inrs.ca (M.A.E.K.); 5 CNR IMM-MATIS, Via S. Sofia 64, 95123 Catania, Italy; simona.boninelli@ct.infn.it; 6 Tekna Plasma Systems Inc., 2935 Industrial Blvd., Sherbrooke, QC J1L 2T9, Canada; Richard.Dolbec@tekna.com

**Keywords:** Si nanostructure, pump-probe, quantum-confinement

## Abstract

To exploit the optoelectronic properties of silicon nanostructures (SiNS) in real devices, it is fundamental to study the ultrafast processes involving the photogenerated charges separation, migration and lifetime after the optical excitation. Ultrafast time-resolved optical measurements provide such information. In the present paper, we report on the relaxation dynamics of photogenerated charge-carriers in ultrafine SiNS synthesized by means of inductively-coupled-plasma process. The carriers’ transient regime was characterized in high fluence regime by using a tunable pump photon energy and a broadband probe pulse with a photon energy ranging from 1.2 eV to 2.8 eV while varying the energy of the pump photons and their polarization. The SiNS consist of Si nanospheres and nanowires (NW) with a crystalline core embedded in a SiO_x_ outer-shell. The NW inner core presents different typologies: long silicon nanowires (SiNW) characterized by a continuous core (with diameters between 2 nm and 15 nm and up to a few microns long), NW with disconnected fragments of SiNW (each fragment with a length down to a few nanometers), NW with a “chaplet-like” core and NW with core consisting of disconnected spherical Si nanocrystals. Most of these SiNS are asymmetric in shape. Our results reveal a photoabsorption (PA) channel for pump and probe parallel polarizations with a maximum around 2.6 eV, which can be associated to non-isotropic ultra-small SiNS and ascribed either to (i) electron absorption driven by the probe from some intermediate mid-gap states toward some empty state above the bottom of the conduction band or (ii) the Drude-like free-carrier presence induced by the direct-gap transition in the their band structure. Moreover, we pointed up the existence of a broadband and long-living photobleaching (PB) in the 1.2–2.0 eV energy range with a maximum intensity around 1.35 eV which could be associated to some oxygen related defect states present at the Si/SiO_x_ interface. On the other hand, this wide spectral energy PB can be also due to both silicon oxide band-tail recombination and small Si nanostructure excitonic transition.

## 1. Introduction

Crystalline silicon is one of the most used material in electronics and photovoltaics. Contrastingly, its use is limited in photonics, owing to its poor optoelectronic properties. Because of its indirect bandgap and the conservation of momentum restriction, bulk silicon has inefficient bulk phonon-assisted emission and absorption of photons. Nevertheless, the nanostructuration of silicon has opened new prospects for the tailoring of its optoelectronic properties. Indeed, silicon nanostructures (SiNS), including porous Si, Si nanocrystals (SiNC) and Si nanowires (SiNW) have attracted a growing interest both from theoretical and experimental perspectives [1]. The size reduction of the Si nanostructures down to reach quantum confinement (QC) conditions permits the manipulation of the silicon band structure. Thus, quantization-related effects increase the bandgap energy and produce a gradual relaxation of the momentum conservation [2], with the consequent increase of the probability of radiative excitonic emission, even if the transition may remain indirect. This means that QC SiNS promise to overcome the bulk crystalline Si poor optical properties due to its indirect bandgap which reduces the efficiency of light emission. In addition, by decreasing the size of the QC SiNS, the photon emission spectrum blue shifts and increases in intensity. Tuning the photon emission of nanosilicon could lead to the development of novel photonic devices [1] and may pave the way to an all-silicon integrated optoelectronics [3,4,5]. Thus, better understanding of the QC effects and charge carrier dynamics in SiNS is essential for the development of nano-silicon based optoelectronic devices.

Time-resolved and stationary photoluminescence (PL) is the most used technique to investigate the QC effects on the electronic structure of SiNS. The majority of reported studies so far has dealt with SiNC. For SiNC embedded in a silicon oxide shell or matrix, the visible (VIS) and near-infrared (NIR) room-temperature PL (up to 2.1 eV) can be ascribed to excitonic recombination with possible participation of oxygen and/or interface related states [6,7,8,9]. For SiNC with diameters less than 3 nm which are expected to emit PL at energies higher than 2.1 eV, no PL blue-shift has been reported so far with the further reduction in SiNC size. This has been interpreted as due to recombination at some oxygen-related defect interface state, which are inside the energy bandgap and entrap both the electron and the holes (self-trapped exciton, STE) [8]. These trap states have a recombination time much faster than that of the radiative excitonic transition. Different oxygen-related defects give rise to different energy states, namely (i) those centered around 2.9 eV (i.e., 420 nm) for the O–Si–O from small SiNC in SiO_2_ matrices and in defect-rich SiO_2_ [10,11], and (ii) those around 2.2–2.4 eV (i.e., 510–550 nm) in the case of the positively charged oxygen vacancies (E’δ) [12]. For SiNW embedded into a silicon oxide shell, only very few papers have been published on the effect of QC on their optoelectronic properties. In particular, QC-induced PL emission has been reported to occur at 1.93 eV (640 nm) for 5 nm-diameter SiNW and to redshift with increasing diameters [13], exhibiting a few tens of μs recombination time [14]. In the recent past, we measured the PL spectra on SiNC and SiNW with diameter down to 2–3 nm, confirming the presence of PL features located between 600 and 950 nm (2.0–1.3 eV) [15]. More recently, we showed that amorphous silicon oxide presents PL in such a spectral range due to band-tail recombination [16].

Complementary to the information gathered through static and time-resolved PL experiments (generally probing the ns-μs time scale), the ultrafast pump-probe transient transmittivity (TT) and reflectance (TR) spectroscopies, investigate the initial charge separation and migration after optical excitation on a femtosecond (fs) time scale [17]. This ultrafast transient spectroscopy is a powerful tool for the study of light emission mechanism and charge-carrier mobility and lifetimes, being sensitive to the sum of the electron and hole population distribution and giving information on both radiative and non-radiative relaxation pathways. Moreover, TT and TR experiments can also provide insights into the nonlinear optical behavior of the material and therefore potentially access useful information for photonic applications. Earlier studies on ensembles of large diameter (>10 nm) SiNW, [18] detected a broadband increase of the SiNW optical transmittivity (the so-called photobleaching, PB). After tens of picoseconds (ps) from the ultraviolet (UV) pump pulse, PB signal is followed by optical absorption (the so called photoabsorption, PA) living a few hundreds of ps. The transient signals were interpreted in terms of filling of conduction states followed by carrier relaxation to lower energy levels in the conduction band and trapping at mid-gap surface defect states. The decrease of the PB lifetime with the SiNW average diameter increase suggests that surface traps are the main channel for carrier recombination [18]. Moreover, the excitation power density dependence of the PB signal dynamics indicates that Auger processes can also play a role in the carrier recombination [18]. Similar results were reported using pump-probe microscopy on isolated SiNW [19,20,21,22] with a diameter between tens and one hundred nm, where the carrier diffusion along the wire axis was spatially and time resolved, allowing the measure of the surface recombination velocity which is found to be lower in silicon oxide coated SiNW [19]. The transient signal between 1.77 and 2.06 eV for large SiNW embedded in silicon oxide shell, presents a PB decreasing with three time-relaxation constants. This result was interpreted as three steps process. First there is an ultra-fast photoelectron population of the conduction band. Then a non-radiative electron decay involving shallow and deep trap states (due to interface defects). Eventually a radiative electron recombination with the hole with a time constant of 75 ps [23]. All the aforementioned works have dealt with intrinsic SiNW having quite large diameters (≥10 nm). For such SiNW diameters, QC effects should not take place and their carrier dynamics and optical bandgaps behave like in bulk silicon. In bulk Si, however, the transient reflectance, is dominated by a long-living negative signal measured in a wide spectral range between 1.1 and 3.0 eV [24,25]. The observation of this negative transient reflectance a few hundreds of fs after the probe has been demonstrated to correspond to a PB driven by the electron transition from the top of valence band to the conduction band [24,25]. On the other hand, negative transient reflectance a few ps after the probe has been mainly ascribed to the changes of the dielectric function induced by the presence of Drude-like free carriers in the conduction band and therefore readable as a signal corresponding to a PA behaviour [24].

In this work, femtosecond time resolved transmittivity spectroscopy has enabled us to shed light on the nature of carrier relaxation pathways in QC SiNS embedded in a few nanometer silicon-oxide shell. The SiNS investigated here mainly consist of spherical and non-isotropic SiNC, which have been separated from the largest spherical Si microstructures by means of a centrifugation based extraction process, as detailed in Ref. [15]. By performing TT measurements with a tunable pump photon energy and a supercontinuum probe pulse (1.2–2.8 eV energy range) as a function of different parameters (i.e., the pump light energy (in the UV and NIR), the light polarization, and high fluence regime), we were able to point out, for the first time, the carrier relaxation mechanism in the ps regime for non-isotropic SiNS. Our results reveal a PA behavior for pump and probe parallel polarizations located between 2.4 and 2.8 eV, which can be associated to non-isotropic ultrafine QC SiNS and ascribed either (i) to the electron promotion from some mid-gap state Si/SiO_x_ interface level to some empty level above the bottom of the conduction band or (ii) to the Drude-like free-carrier presence induced by the direct-gap transition in their band structure. In addition, we found that a broadband and long-living PB in the energy range between 1.2 and 2.0 eV with a maximum in intensity around 1.35 eV. We assign such PB to band tail recombination occurring in silicon oxide coating the Si micro and nanostructures and to small QC SiNS excitonic energy gap transition [16]. The PB maximum could be also ascribed to some defect states present at the Si/SiO_x_ interface [6,25,26].

## 2. Materials and Methods

The SiNS were synthesized using an inductively coupled plasma-torch (from Tekna Plasmas Systems [27]) as a by-product of a spheroidization of Si microparticles [28]. From the process described in refs. [15] and [28], we obtained a condensate consisting of a blend of NW (with diameters in the few nm range and up to several microns long) and silicon spheres with diameters ranging from 10 s of nm to few µm. Aimed by the purpose of selecting the NW population from the silicon spheres, the as-grown (AG) Si nanopowder was dispersed in isopropanol (IPA, Merck KGaA, Darmstadt, Germany) at a concentration of 0.5 mg/mL and ultrasonicated for 5 min in a 200 W ultrasonic bath. The suspension was then centrifuged (Sorvall Legend X1) at a 5000× *g* force for one hour. After centrifugation, the supernatant was taken with a pipette and used to spray-deposit the SiNS onto quartz substrates for pump probe measurements. During spray coating, the substrates were positioned onto a hot plate kept at 80 °C to guarantee rapid evaporation of the IPA. The resulting sprayed nano-Si films were very thin and semi-transparent. The 5000× *g* centrifuged films (hereafter named 5k) were also annealed (for 1 h at 800 °C) under H_2_(5%)/N_2_(95%) forming gas environment at atmospheric pressure [15]. To these centrifuged and annealed films, hereafter we will refer as 5k@.

The structural and chemical characterizations were carried out using a 200 keV JEOL JEM 2010F Transmission Electron Microscope (TEM, Nanolab Technologies, Milpitas, CA, USA) equipped with a Gatan Image Filter to perform Energy Filtered TEM (EFTEM). For TEM analysis purposes, few drops of the supernatant solution were dropped on carbon lacey grids.

The stationary PL measurements were conducted at room temperature by using a 405 nm (3.06 eV) solid state laser excitation line. The incident laser power was 12 mW with a power density of 0.7 W/cm^2^. The emitted light was collected through an optical fiber into a USB5000 Ocean optics CCD spectrometer (Ocean Insight, Largo, FL, USA). A 405 nm notch filter was placed between the laser and the sample and a 475 nm long pass filter was positioned between the sample and the spectrometer to prevent any diffuse reflection from the laser dispersion around the notch filter or simply light passage through the notch filter itself. All PL spectra were corrected for the system response curve.

Transient optical experiments were used to characterize the photo-induced ultrafast phenomena in our nano-Si films spray-coated onto quartz substrates. The carrier distribution was modified by a very intense ultrashort (130 fs) pump beam, while the time evolution of the photoexcited carriers was followed with a broadband probe pulse generated by a sapphire crystal with a photon energy ranging from 1.2 eV to 2.8 eV.

Transient transmittivity measurements were performed in a variable-pump and a supercontinuum-probe configuration by using a 1 KHz amplified Ti:Sapphire laser system capable to supply 0.5 mJ, 150 fs, 1.55 eV light pulses. The pump photon energy can be tuned in two energy regions: between 0.75–1.01 eV and 1.7–2.0 eV by using the output of a traveling wave optical parametric amplifier (TOPAS) and the second harmonic generation through a nonlinear optical crystal. The supercontinuum-probe was obtained by focusing the light beam on a sapphire crystal. The experimental resolution was ΔT/T = 10^−4^, and the probe energy resolution was 0.1 eV.

## 3. Results

### 3.1. Sample Characterization

**Figure 1:**
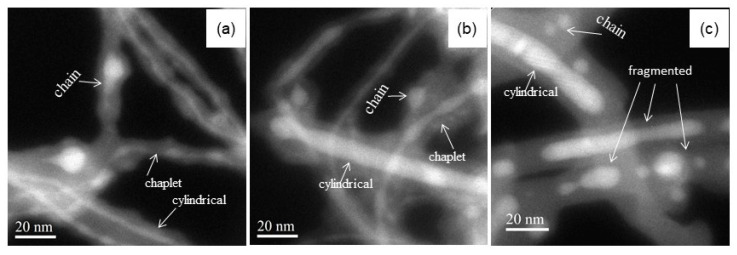
Typical EFTEM images recorded at the plasmon energy loss peak for Si of the as-grown (**a**), centrifuged (**b**) and 800 °C annealed (**c**) Si nanopowders. These EFTEM images reveal the Si core (whitest structures) wrapped in a silicon oxide shell (whitish casing).

The morphologies of both as-grown and centrifuged silicon nanopowder have been investigated in details by scanning electron microscopy (SEM) and TEM, as reported elsewhere [15,29]. The AG silicon nanopowder consists of entangled few-µm-long NW and nanospheres (NSP) with various sizes (from few to 100 nm-diam.). After the centrifugation based purification process, the amount of Si NSP is reduced significantly. Indeed, the ratio of NSP to the other nanostructures composing the sample decreases from the 78% (in the AG powder) to the 15% (in the centrifuged one), while the mean diameter of these Si NSP decreases from 90 nm to 17 nm [15]. The EFTEM studies allowed us to identify the presence for each nanostructure of a Si crystalline core covered by an outer silicon oxide shell a few nanometers thick. The EFTEM also enabled us to identify different types of morphologies inside the NW, namely: (i) NW having a cylindrical continuous core (the real Si nanowires, referred to as SiNW); (ii) NW presenting a modulated Si nanocore consisting of finely connected “almond-shaped” Si nanocrystals, (referred here to as “chaplet-like” NW) and (iii) NW of which core exhibits disconnected spherical Si nanocrystals (here named “chain”). The same three Si crystalline core morphologies are present in both AG and centrifuged samples, as unveiled by the whitest features displayed in Figure 1a,b. Figure 1, indeed, reports EFTEM images acquired at 17 eV, i.e., the energy corresponding to the plasmon energy loss feature of Si. In such images, the higher is the Si atom density, the whiter is the color. After the AG powder annealing at 800 °C in forming gas for 1 h, a new morphology appeared (Figure 1c, whitest structures) where the Si core is found to be fragmented in drops of different lengths and shapes (i.e., cylindrical or oblate) or locally strangled [30]. We will refer to all these nano-silicon morphologies as fragmented SiNW. Notably, all these fragmented SiNW have a non-isotropic shape. In this case, no chaplet-like NW was detected.

**Figure 2:**
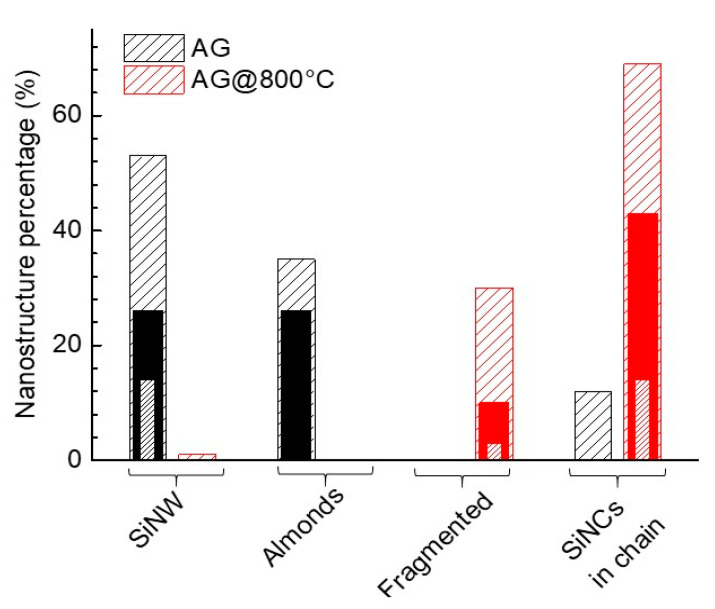
Percentage of SiNW, Almonds, Fragmented SiNWs and SiNCs in chain (sparse diagonals), percentage of each type of SiNS with diameter d ≤ 5 nm (solid field) and with d ≤ 3 nm (dense diagonals) for the AG (black) and AG@800 °C (red).

Figure 2 reports the SiNS morphology distribution (sparse diagonals) and the percentage of them with respect to the total amount of elongated nanostructures having a diameter less than 5 nm (solid) and in the 2–3 nm range (dense diagonal) for the AG (black) and annealed (red) nanopowders. Interestingly, by evaluating the percentage of SiNS with diameters between 2 and 3 nm with respect to the total number of elongated structures, this percentage increases from 14% to 17% as a consequence of the annealing. However, this latter percentage decreases to 3% if we consider only the non-isotropic Si nanostructures. Therefore, summarizing we can say that: (1) the centrifugation process removes the large diameter Si micro- and nanospheres, thus increasing the percentage of the NW with respect to the total number of the Si structures; (2) due to the annealing procedure the percentage non-isotropic SiNS with respect to the total number of the elongated structures reduces from 88% to 30%. The percentage of those with diameter <5 nm decreases from 52% to 10% (see Figure 2) and further lessens from 14% to 3% when considering only species with diameters in the (2–3) nm range.

**Figure 3:**
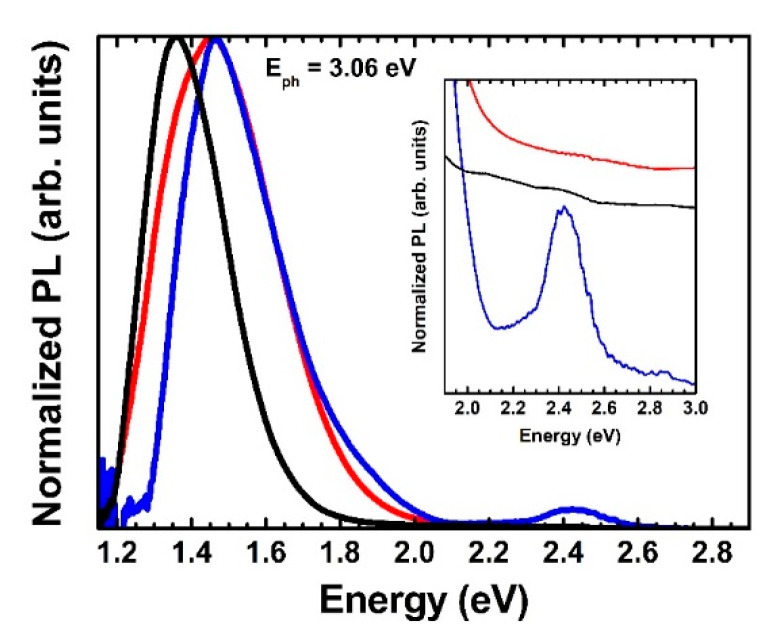
Static PL normalized spectra of the as grown (AG, black curve), 5000× *g* centrifuged (5k, blue curve) and 5000× *g* and annealed in forming gas at 800 °C (5k@, red curve). The inset is a zoom of the 1.9–2.9 eV spectral region.

**Figure A1:**
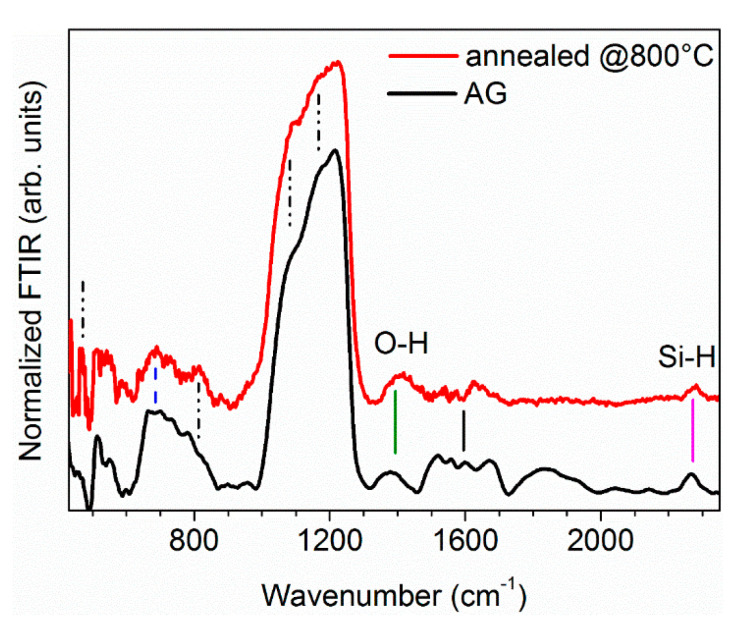
FTIR spectra of as-grown (AG) (black curve) and annealed (red curve) samples.

Figure 3 shows the static photoluminescence (PL) spectra of: (i) the AG Si nanopowder (black curve); (ii) the 5000× *g*-centrifuged sample (blue curve); and (iii) the 5000× *g*-centrifuged and annealed in forming gas at 800 °C (red curve) samples. The PL spectra have been normalized so to have an equal PL value at their most intense feature. For all the three spectra two features are visible, namely the most intense peak, ranging for all the samples between 1.2 eV and 2.0–2.1 eV and a weaker peak located in the 2.0–2.8 eV energy range. The AG PL most intense peak is centered at 1.36 eV, has a full width at half maximum (FWHM) of 0.26 eV and it is slightly asymmetric, presenting a weak tail arriving at about 2.0 eV. The most intense PL peaks for both the 5k and 5k@ samples have their maximum around 1.46 eV, a relatively large FWHM (0.30 eV and 0.37 eV, respectively) and are asymmetric (the former presenting a shoulder around 1.3–1.4 eV while the latter showing a shoulder around 1.9 eV). The 1.2–2.1 eV range PL features can be assigned to the various small-diameter SiNS present in the Si nanopowder samples [15] as well as to the presence of silicon-oxide coating the SiNS due to band tail recombination [16]. Indeed, the energy gap of quantum confined spherical SiNC and SiNW with d ≤ 5 nm is theoretically expected to be greater than 1.45 eV and 1.26 eV, respectively [26]. Therefore, the PL blue-shift and asymmetric broadening after the purification process indicates that the centrifugation process could reduce the percentage of the biggest QC SiNS having an intense PL at room temperature with respect to the total number of radiatively emitting SiNS. On the other hand, a PL emission around 1.35 eV is reported to be due to defect states or self-trapped excitons at the SiOx/Si interface [6,25] or in Si-Si dimers at the nanostructure boundaries [31]. Such considerations suggest that (i) the purification removes a high content of silicon oxide species, which happens because of the high reduction of large micro and nano Si spherical particles coated by a few nanometer thick silicon oxide; (ii) the annealing can modify the silicon oxide amount covering the SiNS, since the 5k@ film displays a shoulder located around 1.35 eV which is absent in 5k sample. As for the PL features in the 2.0–2.8 energy range, they can be associated to oxygen-related interface states [10,11,12]. Indeed, for silicon oxide coated Si nanostructures smaller than 3 nm-diam., no PL emission from indirect gap energy is expected in this region due to the presence of oxygen-related interface states [8] in the gap of these ultra-small nanostructures and their consequent participation in the PL process [7]. We defined the ratio, R_PL,_ between the PL area underlying the 2.0–2.8 eV and the total energy range. It can be associated to the percentage of ultra-small nanostructures with respect to the total amount of the PL generating species, including QC Si nanostructures and oxygen-related interface states in the gap. The R_PL_ value is 0.004, 0.016 and 0.004 in the AG, 5k and 5k@ films, respectively. The former increase confirms the effectiveness of the purification process in increasing the percentage of the ultra-small Si nanostructures. At the same time, the R_PL_ reduction as a consequence of the annealing suggest that there should be a partial oxygen-related state healing as reported in ref. [16] and shown in the FTIR spectra of Figure A1 in the Appendix A. Such a partial healing is able to reduce the PL intensity due to oxygen-related interface states inside the ultrasmall SiNS bandgap but not to completely eliminate them thus limiting much the PL emission from QC SiNS excitonic recombination.

### 3.2. Pump-Probe Measurements

**Figure 4:**
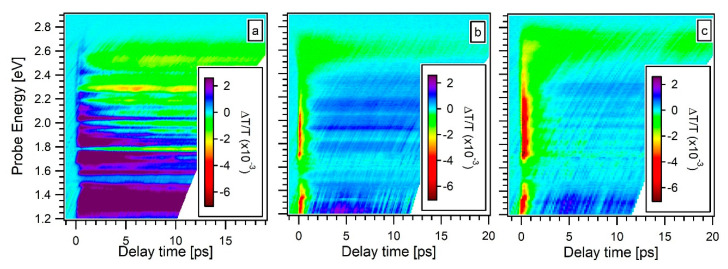
Transient transmittivity ΔT/T (E,t) diagrams of the AG (**a**), the 5k (**b**) and 5k@ (**c**) samples. In the inset a color scale indicates the transient transmittance intensity.

**Figure 5:**
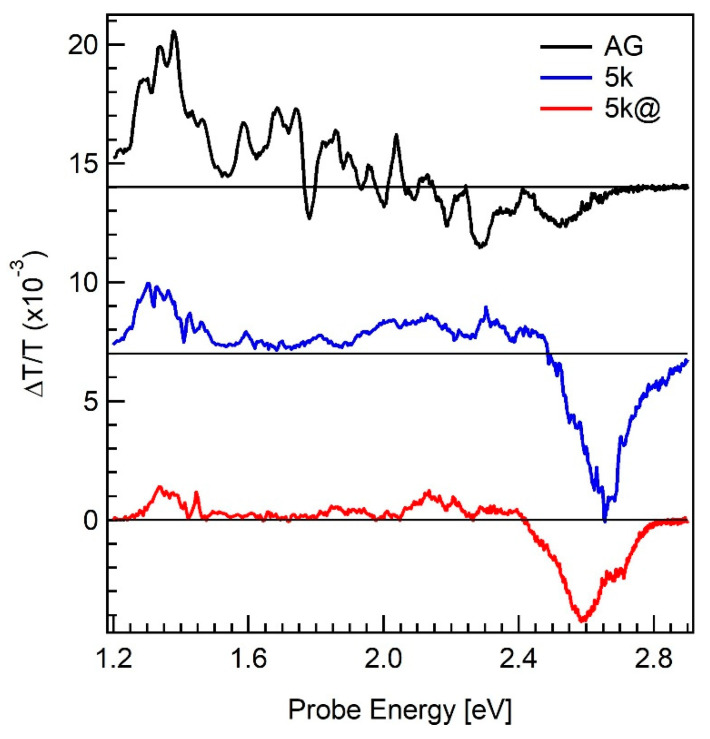
Transient transmittivity ΔT/T (E, t = 5 ps) curves at long dynamics collected on the AG (black), the 5k (blue) and the 5k@ (red) samples. All the measurements are performed with a pump photon energy of 3.27 eV and with an excitation fluence of 3.5 mJ/cm^2^. Both pump and probe pulses are linear polarized and mutually parallel.

Figure 4 shows the transient transmittivity ΔT/T (E,t) diagrams collected on the AG (Figure 4a), the 5k (Figure 4b) and 5k@ (Figure 4c) samples. All the measurements were performed with a pump photon energy of 3.27 eV and an excitation fluence of 3.5 mJ/cm^2^. Both pump and probe pulses are linearly polarized and mutually parallel. A positive ΔT/T (i.e., photobleaching, PB) indicates that the probed states are occupied by free-carriers or excitons, and a negative ΔT/T (i.e., photoabsorption, PA) is indicative of an absorption of probe photons promoting the valence electrons to empty states. The ΔT/T (E,t) diagram of the AG samples (Figure 4a) is seen to present a broad photobleaching (PB) transient, starting from the zero-delay condition and evolving in a multi-picosecond PB band, located in the probe photon energy range between 1.2 eV and 2.0 eV. Both the 5k (Figure 4b) and 5k@ (Figure 4c) samples exhibit similar ΔT/T (E,t) diagrams. They both present an initial PA broadband followed by a PB transient located in the 1.2 eV–2.4 eV range. The initial PA is ultrafast, much less than 1 ps, and is the typical contribution of the quartz substrate. The quartz PA contribution is visible for these two films because of their relatively small thickness (very high optical transparency) in comparison with the AG sample. A long-lived PA band, localized between 2.4 and 2.8 eV, is also present. All the ΔT/T (E,t) diagrams show an additional modulation in the signal magnitude dependent only on the probe photon energy. This modulation, which visually manifests as horizontal stripes in the data matrices (Figure 4), originates from the interference fringes due to the thickness of the film. Such an assignment is confirmed by the variation of the modulation with the probe incidence angle and with the sample position (data not reported here), as expected for an interference phenomenon which mainly depends on the sample thickness.

**Figure A2:**
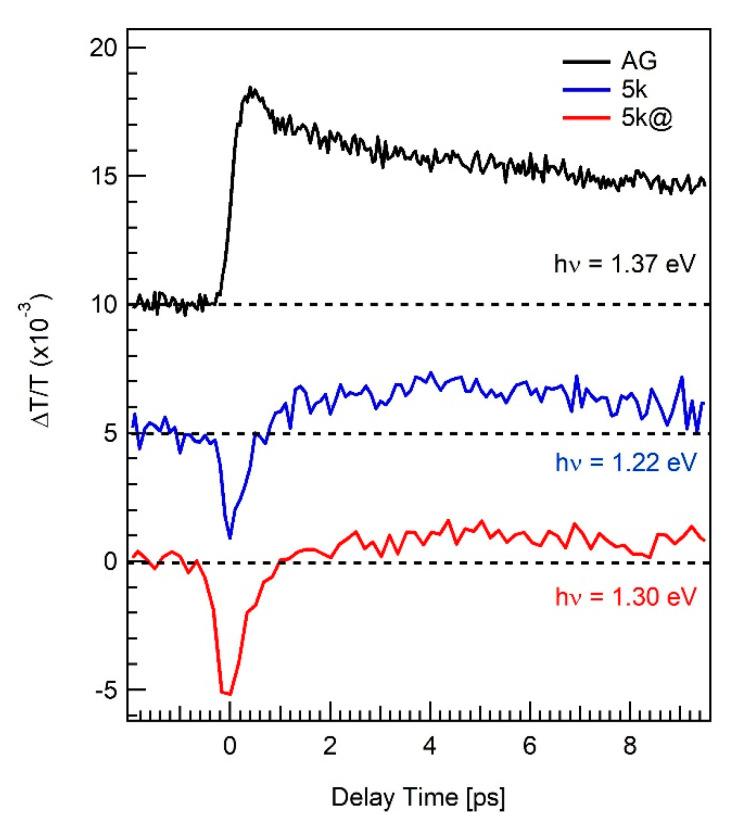
Behavior of the transient transmittance versus the pump and probe delay time collected on the AG (black), the 5k (blue) and the 5k@ (red) samples. ΔT/T profiles are taken at the maximum of the PB band (Figure 4 of the article).

Figure 5 compares the ΔT/T curves at long dynamics (E, t = 5 ps) collected at a pump-probe delay of t = 5 ps, for the AG (black line), 5k (blue line), and 5k@ (red line) samples. In addition to the interference fringes, one additional positive band, indicating a PB process, is present in all the samples (Figure 5, black line) between 1.2 and 2.0 eV and featuring a maximum at ~1.35 eV. A second band, centered at ~2.6 eV, is clearly visible for the 5k and 5k@ samples, which being negative is related to a PA process. As previously mentioned, a PB process is generally due to the presence of photocarriers in the probed energy level. This means that, for all the samples, there are energy levels between 1.2 and 2.0 eV, occupied by excited photocarriers just after 5 ps from the pump pulse. Actually, as visible by inspecting the behavior of the transient signal as a function of the pump-probe delay time registered at fixed probed energy (see the color evolution along a horizontal axis in Figure 4a), such a relaxation time is even shorter (~1 ps). Moreover, the photocarriers live in those levels for a long time, since a very small attenuation of the PB signal is registered over time (see Figure 4a and Figure A2 of the Appendix A). In the case of SiNS, the former time scale is slower than the one expected for the relaxation time at the conduction band edge of the excited electrons through phonon (ph) emission in the case of bulk Si (of the order of fs to ps) because of the reduced amount of phonon modes due to the discretization of phonon density of states due to QC effects [32]. However, since the impinging photon flux is very high, it is reasonable to suppose a corresponding increase in the phonon number giving rise to an increase of e-ph scattering events which translates in a shortening of the relaxation time of the photoexcited electrons to the bottom of the conduction band. On the other hand, our time-scale is too short to allow us to distinguish among the four possible e-h recombination types: i.e., radiative decay through discrete defects or interface levels (in the nanosecond range), radiative decay between the bottom of the conduction band and defect or interface level, radiative SiNS interband recombination (of the order of μs), radiative silicon oxide band tail recombination. Actually, due to the high incident photon flux, it is not possible to exclude the coexistence of all these radiative decay pathways. Nonetheless, the extension of PB transient signal over the same wide range of the PL feature (i.e., between 1.2 and 2.0 eV) makes highly reliable that two of the origins of the PB transient signal are the exciton recombination occurring at the several energy bandgap of the nanometer-sized QC SiNS and band tail recombination arising from the amorphous silicon oxide coating the Si micro and nanostructures [16]. Furthermore, it is worth noting that the maximum of PB for all the three samples is always centered around 1.35 eV, while the PL maximum after the centrifugation process resulted blue-shifted to 1.46 eV. This suggests that the origin of this maximum could be due to defect states or self-trapped excitons at the SiOx/Si interface [6,23] or in Si-Si dimers at the nanostructure boundaries [31]. SEM and EFTEM analyses suggest that, as a consequence of centrifugation, we have a strong reduction of the very large diameter SiNS. These SiNS are expected to behave in terms of PL as a bulk Si and therefore with negligible PL at room temperature. On the other hand, the purification, by decreasing the percentage of the large Si spherical particles with respect to the total amount of SiNS, has hugely reduced also the relative contribution of these interface defect states. This effect was also observed in the static PL.

**Figure 6:**
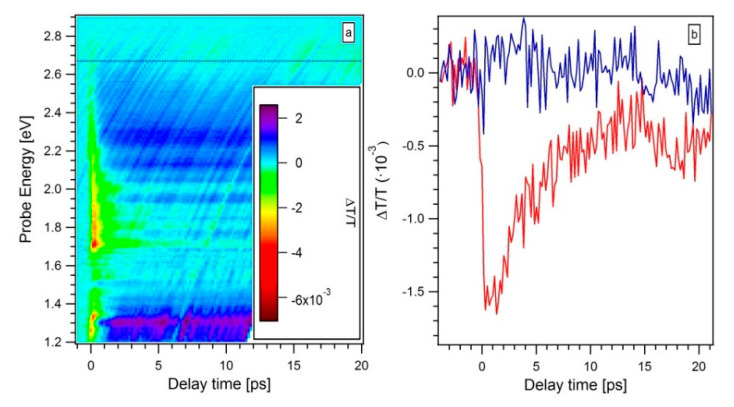
(**a**) Transient transmittivity ΔT/T (E,t) collected on the 5k@ film where the pump and probe pulses are linearly polarized and mutually perpendicular. The inset shows a color scale intensity for the transient transmittance. (**b**) Comparison of the transient transmittance ΔT/T (E = 2.66 eV,t) acquired at a fixed energy of the probe on the 5k@ sample with the pump and probe pulses linearly polarized and mutually parallel (red curve) and perpendicular (blue curve). All the measurements are performed with a pump photon energy of 3.27 eV and with an excitation fluence of 3.5 mJ/cm^2^.

Considering the great inhomogeneity, both in shape and dimensions of the nanostructures in the films and the differences in film thicknesses, it is of no meaning to compare the intensity of the PB maximum among the three samples. However, it is worth evaluating the relative ratio R between the area of PA and PB bands for each film. R results to be 2.1 and 7.7 for the 5k and 5k@ films, respectively. The PA band in the AG sample can be considered absent, within the measurement noise. This means that the R value in the AG case is near to zero. Such a significant increase of the R value after the centrifugation, suggests that the PB can be correlated to larger Si micro- and nanostructures, which the centrifugation process removed from the 5k and 5k@ samples. This assumption is consistent with the interpretation that associates the maximum of the PB to the radiative recombination involving energy levels at the oxide/Si interfaces. Indeed, its weight with respect to all the other recombinations, drastically reduces (as a consequence of the significant decrease of the interfaces of the largest Si micro- and nanostructures). A further increase of R is then observed after the annealing, which indeed decreases the density of oxygen vacancies in the samples as shown by the Fourier Transform Infrared data reported in Figure A1 of Appendix A. PA signal is normally interpreted as the absorption of probe photons promoting electrons from some transiently filled energy states (usually the conduction band or some short-living intermediate state) to empty states. In our case, the most relevant PA band after 5 ps is observed between 2.4 and 2.8 eV. The long living PA signal can be interpreted as an absorption transition induced by the probe toward some empty level whose onset is the valence band itself or some short-living intermediate state at the oxide/Si core interface. These empty levels could be identified in the interface defect states and in the bottom of the conduction band for the ultra-small nanostructures presenting an indirect band-gap energy between 2.4 and 2.8 eV. Figure 6a shows a typical ΔT/T (E,t) diagram of the 5k@ sample, with mutually perpendicular pump and probe polarizations. The measurement is performed on the same sample point of the ΔT/T (E,t) diagram shown Figure 4c. The pump fluence is kept at of 3.5 mJ/cm^2^ and tuned at a photon energy of 3.27 eV, only its polarization plane is rotated by 90° with respect to the previous case (Figure 4c). From the comparison of the two ΔT/T (E,t) diagrams (Figure 4c and Figure 6a), the strong dependence of the PA signal intensity on the relative pump and probe polarization direction is evident. Whilst the PA magnitude at zero-delay time (which we associate to the quartz substrate) does not considerably change in the crossed polarization case (compare the color scale in Figure 6a and Figure 4c), the long lived PA band between 2.4 and 2.8 eV is totally suppressed in the whole delay time window. Figure 6b compares the temporal evolution of the ΔT/T signal collected at 2.66 eV for both the cross-polarized (blue curve) and parallel-polarized (red curve) pump and probe configurations. The signal magnitude of the PB band, located in the 1.2 eV and 2.0 eV energy range, does not vary significantly between the two polarization configurations. The same striking behavior of the PA band is observed on the 5k samples as well.

**Figure 7:**
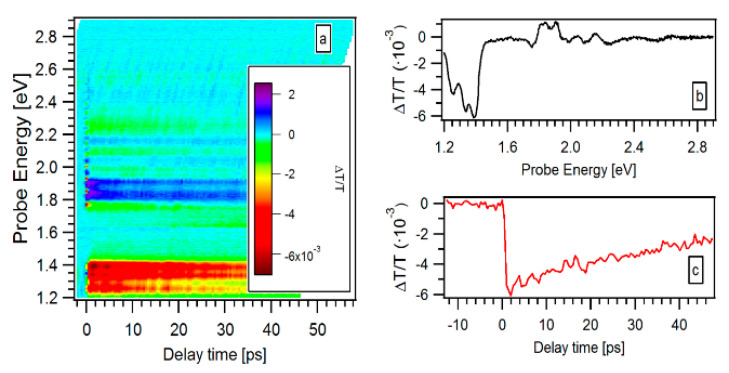
(**a**) Transient transmittivity ΔT/T (E,t) collected on the 5k film with a pump photon energy of 1.63 eV and with an excitation fluence of 7.4 mJ/cm^2^. In the inset a color bar indicates the transient transmittance intensity. (**b**) Transient transmittance ΔT/T (E,t = 5 ps) at long dynamics and (**c**) transient transmittance ΔT/T (E = 1.36 eV,t) at fixed energy of the probe.

The strong polarization dependence of the PA signal (at E = 2.66 eV) suggests that this feature has to be ascribed to asymmetric nanostructures like SiNW, the almonds in “chaplet-like” NWs and fragments inside the fragmented-NWs. This can be understood in terms of classical electromagnetic theory, which predicts a reduction of the electric field inside the nanowire by increasing the angle between the incident electric field and the nanowire axis and for nanostructures with length comparable to or lower than the incident radiation wavelength [33,34]. Since both the probability of optical absorption and emission is proportional to the square of the electric field inside the nanowire, it is likely that the pump will excite, among all the randomly distributed nanowires, only the ones with the axis almost parallel to its electric field. However, in case of cross-polarized pump and probe, those nanowires excited by the pump, will present a strongly reduced absorption when excited by the probe, thus giving rise to a hugely reduced or a zero transient signal. To summarize, it looks that prevalently the non-isotropic SiNS affects the behavior of the PA features between 2.4 and 2.8 eV. Figure 7 reports the transient transmittivity of the 5k sample for an excitation energy in the VIS region (1.63 eV). A similar behavior has been measured for the 5k@ film. By using such an excitation energy, the samples exhibit a lower static absorption than in the UV pump signal, so that in this measurement the pump fluence was increased and kept at 7.4 mJ/cm^2^. Figure 7a reports the obtained ΔT/T (E,t) diagram, at the excitation energy of 1.63 eV, showing mainly a PA transient signal between 1.2 and 1.4 eV probe energies. No traces either of the PA signal are found between 2.4 and 2.8 eV or of the PB band between 1.2 and 2.0 eV. This behavior is better highlighted in the ΔT/T (E,t = 5 ps) profile, displayed in Figure 7b. The temporal evolution of the PA transient is also reported in Figure 7c for a probe energy of 1.36 eV, where the signal magnitude is maximum. The overall shape of the ΔT/T (E,t) signal excited at 1.63 eV remains the same for all the three types of samples investigated here and does not present a strong dependence on the pump and probe relative polarizations (data not reported here). Such a multi-picosecond PA transient (Figure 7c) closely tracks the usual behavior of bulk crystalline silicon after a few ps, rationalized in terms of the Drude-like absorption of the photoexcited electron-hole plasma [24]. Our time resolution is not enough to observe PB signal expected to occur for QC SiNS with energy gap lower than 1.63 eV, in the first hundreds of fs [24,25]. Moreover, if the PA signal in the 2.4–2.8 eV energy range were due to the absorption from the valence band or an interface defect intermediate level to an empty defect state, it should be present even by using a VIS excitation energy. This makes us confident to consider such a PA as due to small-sized QC non-isotropic SiNS, presenting an energy gap in the 2.4–2.8 eV range. These SiNS, having a diameter between 2 and 3 nm, are indeed present in both the 5k and 5k@ films. Another possible interpretation of this PA signal, after a few ps from the probe, could be given in terms of observation of Drude-like absorption of the photoexcited electron-hole plasma induced by the direct band-gap of the non-isotropic QC SiNS. Indeed, it has been recently shown that in bulk Si the transient reflectance, after a few hundreds of fs from the probe, presents a minimum in correspondence of the optical direct gap transition with at Γ point in the Brillouin zone (3.3 eV), thus demonstrating the possibility to induce direct-gap transitions [25]. In addition, it has also been reported that the direct gap in QC Si nanocrystals decreases from 3.0 to 2.2 eV for diameters between 1 and 2.5 nm and increases from 2.2 eV to 3.0 with diameter increasing up to 5.5 nm [35]. In this case, our experiment time resolution could be not enough to evidence the PB signal which is expected to occur in the first one or two hundreds of fs [24].

## 4. Conclusions

In conclusion, we studied the transient transmittivity of ultra-thin films made from ICP-synthesized silicon nanopowder, where Si nanospheres, long and fragmented SiNW, “chaplet-like” nanowires and chains of spherical SiNC are present. From the comparison of as-grown, centrifuged and centrifuged-plus-annealed Si nanopowder samples, using both parallel and cross-polarized pump and probe beams, we succeeded in evidencing the first ps carrier relaxation mechanism of anisotropic ultra-small SiNS (i.e., SiNWs, Si “chaplet-like” and/or fragmented nanowires). Our results clearly reveal a PA behavior for pump and probe parallel polarizations located at about 2.6 eV, which can be associated to non-isotropic ultra-small QC SiNS and interpreted to be due to the multipicosecond occupation of the high energy levels close to the Γ point in their band structure or as an electron absorption induced by the probe from some intermediate mid-gap state toward some empty state above the bottom of the conduction band. Finally, we were also able to distinguish, in the lower energy part of the fast-transient signal, the contributions of some oxygen related defect states, of amorphous silicon oxide band tail recombination and of the excitonic recombination due to small QC SiNS. It is worth noting that the ultrafine size of SiNS and their related band structure contributed to the observed PA and PB behavior. The knowledge of such charge carrier relaxation times and of the mechanism at their origin is fundamental for the exploitation of these ultra-small, silicon-oxide coated SiNS in optoelectronic devices.

## Appendix A

Figure A1 reports the Fourier Transform Infrared (FTIR) spectra of the AG (black curve) and the sample (red curve) annealed at 800 °C for 1 h under H_2_ (5%)/N_2_ (95%) forming gas environment at atmospheric pressure. Both the spectra have been arbitrarily normalized to the about 1220 cm^−1^ peak.

The two spectra show peaks distinctive of the vibration modes of silicon oxide: i.e., the on-phase stretching (1082 cm^−1^), the out-of-phase stretching (1177 cm^−1^), the bending (812 cm^−1^) and rocking (458 cm^−1^) of Si-O-Si (see the dash-dot-dot black lines in Figure A1) [12,36]. However, for stoichiometric SiO_2_, the ratio, R_O_, between the absorption strength of the out-of-phase and in-phase features is 0.43 while it increases for Si-rich oxides [36]. Therefore, our FTIR data indicate that the silicon oxide coating the Si nanostructures is mainly a Si-rich oxide (R_O_ = 1.27) and that with annealing O atoms partially redistribute and the amount of SiO_2_ increases (R_O_ = 1.02). This is also confirmed by the reduction of the strength of the bands around 660–700 cm^−1^ (dashed blue line in Figure A1) and between 1500 and 1750 cm^−1^ (solid black line in Figure A1) which are respectively associated to interstitial O in the Si-Si stretching [37] and to interstitial oxygen [38] vibrations. No sensible variation of the absorption peaks due to O-H located around 1400 cm^−1^ (green solid line in Figure A1) and Si-H bonds about 2270 cm^−1^ (solid magenta line in Figure A1) is observed.

In Figure A2 the behavior of the transient transmittance versus the pump and probe delay time is reported. ΔT/T profiles are taken at the maximum of the PB band of Figure 4 of the article for each sample. For all the samples, the PB feature presents a multi-picosecond relaxation dynamic, which can be ascribed to long living photocarriers in the probed states. They can be associated either to defect state or excitonic transitions which results to relax towards ground state in time scale well longer than tens of ps. The initial PA for the 5k and 5k@ samples is ultrafast, well less than 1 ps, and it is the typical contribution of the quartz substrate, visible for these two films because of their small thickness (very high optical transparency). Unfortunately, it prevents a good analysis of the first ps process. However, the ultra-fast rising of the transient transmittance (less than 1 ps) for the AG sample is compatible both with occupation of defect states and with ps thermalization time of the photocarriers at the bottom of the conduction band for big (micrometer down to tens of nanometer in diameter) Si structures which represent the majority of the AG film [10].
